# A Novel Technique for Labral Reconstruction Using Long Head of Biceps Tendon: Duru Technique

**DOI:** 10.7759/cureus.13254

**Published:** 2021-02-10

**Authors:** Baver Acar, Ozkan Kose, Cihan Kircil, Kerem Canbora, Mehmet Demirtas

**Affiliations:** 1 Orthopaedics, University of Health Sciences, Antalya Education and Research Hospital, Antalya, TUR; 2 Orthopaedics and Traumatology, Department of Orthopaedics, Memorial Ankara Hospital, Ankara, TUR; 3 Orthopaedics and Traumatology, Uskudar University Medical Faculty, İstanbul, TUR

**Keywords:** shoulder dislocation, shoulder instability, duru technique, laterjet, shoulder, long head of biceps tendon

## Abstract

Arthroscopic capsulolabral repair is a well-established surgical treatment for traumatic anterior shoulder instability. When there is insufficient labral tissue during arthroscopic primary or revision Bankart repairs, various soft tissue procedures have been recommended. All these procedures aim to reattach glenohumeral ligaments to the glenoid rim and regain the tight anterior structures to prevent re-dislocation or subluxation. Some authors recommend the Latarjet procedure, even in the absence of critical bone loss in this patient group. The labrum increases the depth of the glenoid cavity, thereby, increasing the glenoid track. It behaves like a block for the humeral head. Reconstruction of the labral tissue may strongly contribute to shoulder joint stability when it is totally absent. In this article, we describe a novel labral reconstruction technique (Duru technique) using the long head of the biceps tendon in two patients without an existing labral tissue.

## Introduction

Arthroscopic capsulolabral repair is a well-established surgical treatment for traumatic anterior shoulder instability, yielding satisfactory clinical results and enabling a return to sports. However, in the case of bone loss exceeding 25% of the glenoid fossa, soft tissue procedures have high failure rates. In these cases, it is necessary to address the bone loss, and the Bristow/Latarjet procedure is usually advocated [1].

After recurrent shoulder dislocations, not only the glenoid bone but also the labrum, capsule, and anterior glenohumeral ligaments may be severely damaged. Franceschi et al. [2] classified labral injury into three types with an arthroscopic examination. According to this classification: type 1 (robust; when there was a discrete labral tissue with the thickness being the greater part of the labrum), type 2 (thinned; when the thickness of the labral tissue was less than half, type 3 (none; when there was no discernible labral tissue).

When there is insufficient labral tissue during the arthroscopic primary or revision Bankart repairs, various soft tissue procedures such as subscapular augmentation, rotator interval narrowing, and capsular plication and shift have been recommended. All these procedures aim to reattach the glenohumeral ligaments to the glenoid rim and regain the tight anterior structures to prevent re-dislocation or subluxation. However, these procedures may result in a restriction in the external rotation of the shoulder joint. Some authors recommend the Latarjet procedure, even in the absence of critical bone loss in this group of patients [3-6].

The labrum is a fibrocartilaginous tissue that increases the depth of the glenoid cavity and increases the glenoid track. Furthermore, it behaves like a block for the humeral head. Reconstruction of the labral tissue may strongly contribute to shoulder joint stability when it is totally absent. In this article, we describe a novel labral reconstruction technique (Duru technique) using the long head of the biceps tendon (LHBT) in two patients who had normal glenoid bone stock without an existing labral tissue.

## Technical report

Description of the surgical technique

**Figure 1:**
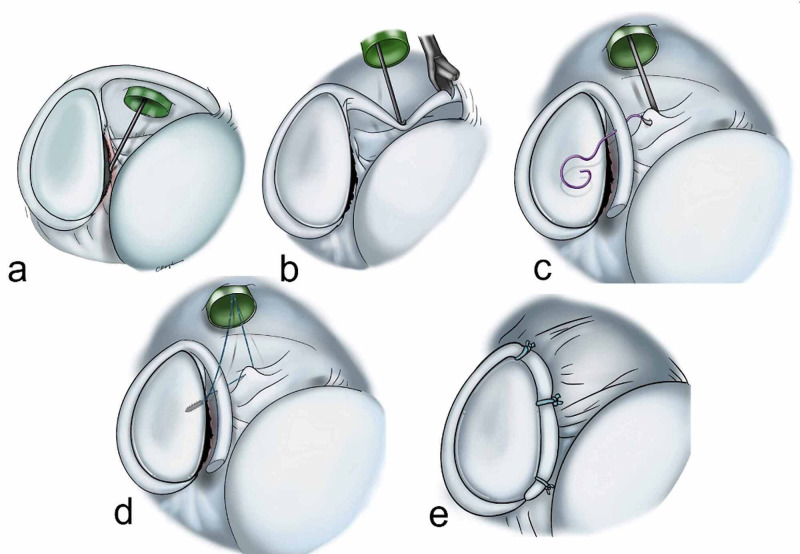
Schematic drawing of the surgical technique. (a) Franceschi type III labral defect; (b) pressure was applied downward with a probe from the anterior portal to ensure a sufficient length of LHBT within the joint for labral reconstruction, and the LHBT was cut with a punch; (c) to ensure capsular tension, the capsule was sutured to the labral tissue; (d) the anchor was placed at the level of 1 o’clock in the location of the biceps and two more anchors were placed at 5 o’clock, and then, 3 o’clock, and labral reconstruction was applied; (e) postoperative intraarticular view. LHBT: long head of the biceps tendon.

With the patient in the lateral decubitus position, an intra-articular approach was used through a standard posterior portal (soft spot). Standard diagnostic arthroscopy was performed with a 300 arthroscope and a pump maintaining pressure at 50 mmHg. Anterior portals were, then, established using the outside-in technique through a spinal needle as a guide. To increase the healing of the soft tissue, the anterior section of the glenoid was prepared with an arthroscopic burr and rasp. The anteroinferior portal (5 o’clock) was, then, opened with the outside-to-inside technique. The pressure was applied downward with a probe from the anterior portal to ensure a sufficient length of LHBT within the joint for labral reconstruction, and the LHBT was cut with a punch (from the anteroinferior portal). First, the suture anchor was placed at the level of 1 o’clock in the location of the biceps and two more suture anchors were placed at 5 o’clock and then, 3 o’clock, and labral reconstruction was applied. To increase capsular tension, the capsule was sutured to the labral tissue, particularly the inferior glenohumeral ligament (IGHL; Figure 1).

Case 1

**Figure 2:**
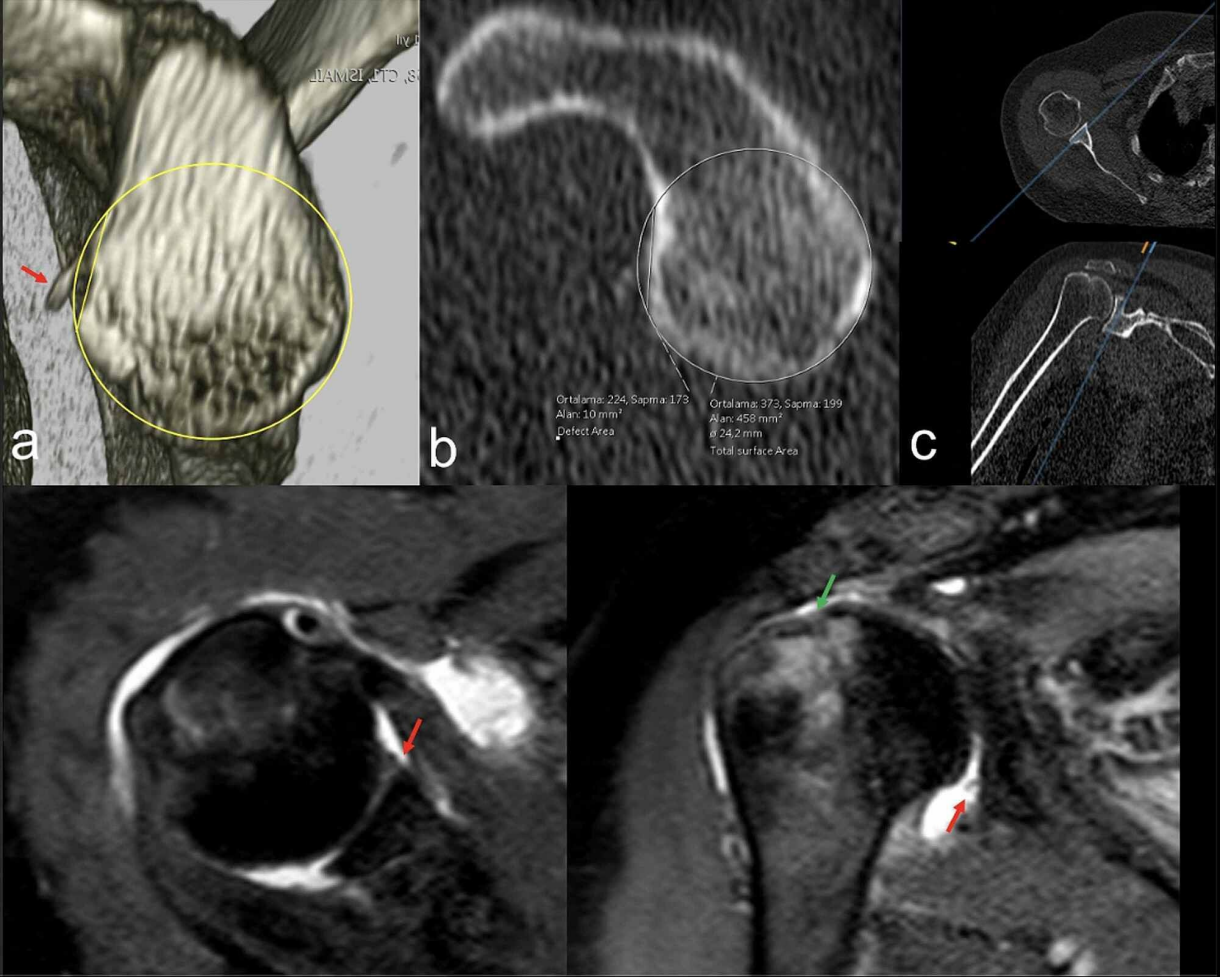
Preoperative images of case 1 (a) Three-dimensional CT image of glenoid; (b) measurement of the bone Bankart defect; (c) MPR images; (d) axial MRI showing defective labrum; (e) Hill-Sachs defect (green arrow) and labral defect (red arrow). CT: computed tomography; MRI: magnetic resonance imaging; MPR: multiplanar reformation.

A 61-year-old female presented to our clinic with complaints of recurrent shoulder dislocations. Her medical history revealed a low-energy trauma one month ago and admission to emergency service with shoulder dislocation. At that time, shoulder reduction was performed; however, she suffered from recurrent dislocation twice a month. She had no prior shoulder injuries before the trauma. On physical examination, the apprehension test and fear test were positive. Computed tomography (CT) revealed 4% glenoid bone loss (GBL). Magnetic resonance imaging (MRI) showed a Hill-Sachs, labral tear, and rotator cuff tear (Figure 2). After the diagnosis of rotator cuff tear and Bankart lesion, arthroscopic labrum and rotator cuff repair was planned. Written informed consent was obtained from the patient.

**Figure 3:**
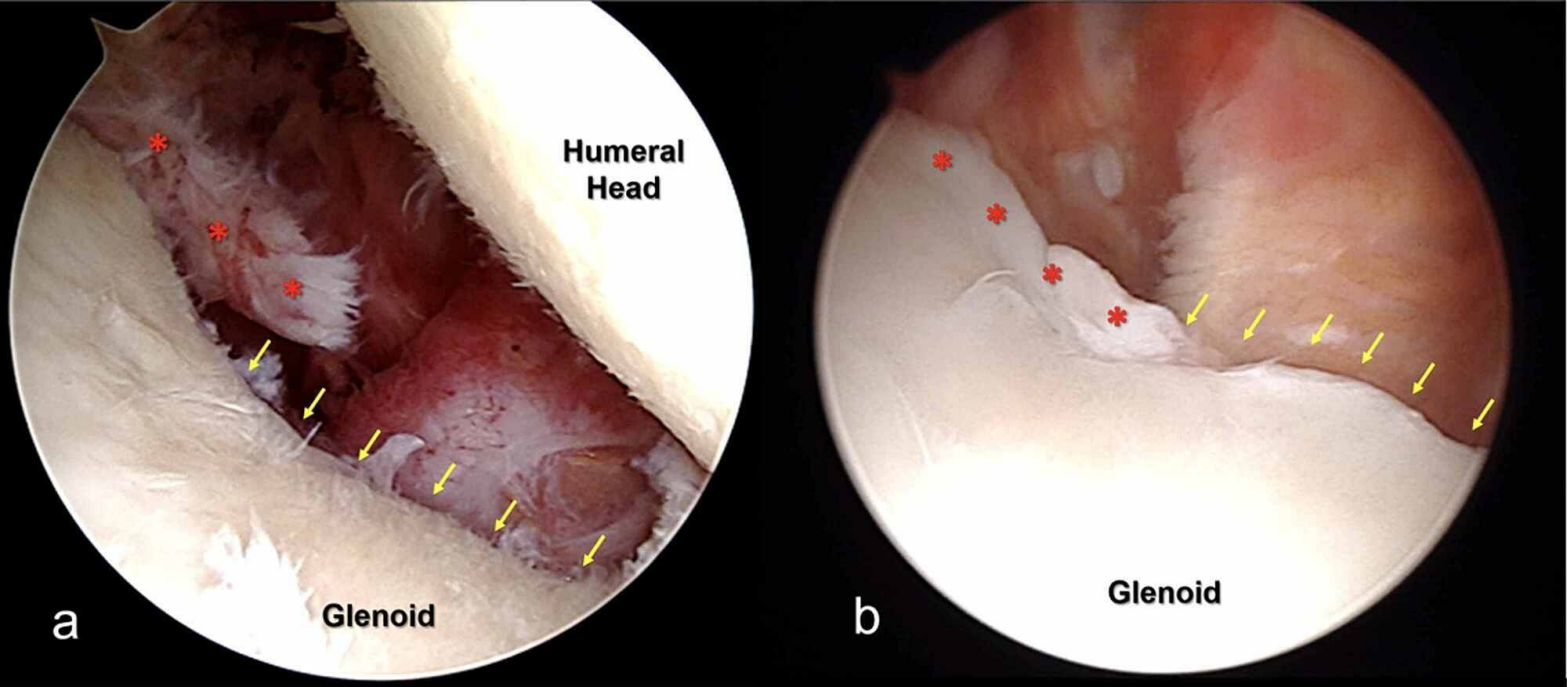
Intraoperative views of type III labral defects Red dots show labral tissue remnant and yellow arrows show there was no discernible labral tissue: (a) case 1 and (b) case 2.

**Figure 4:**
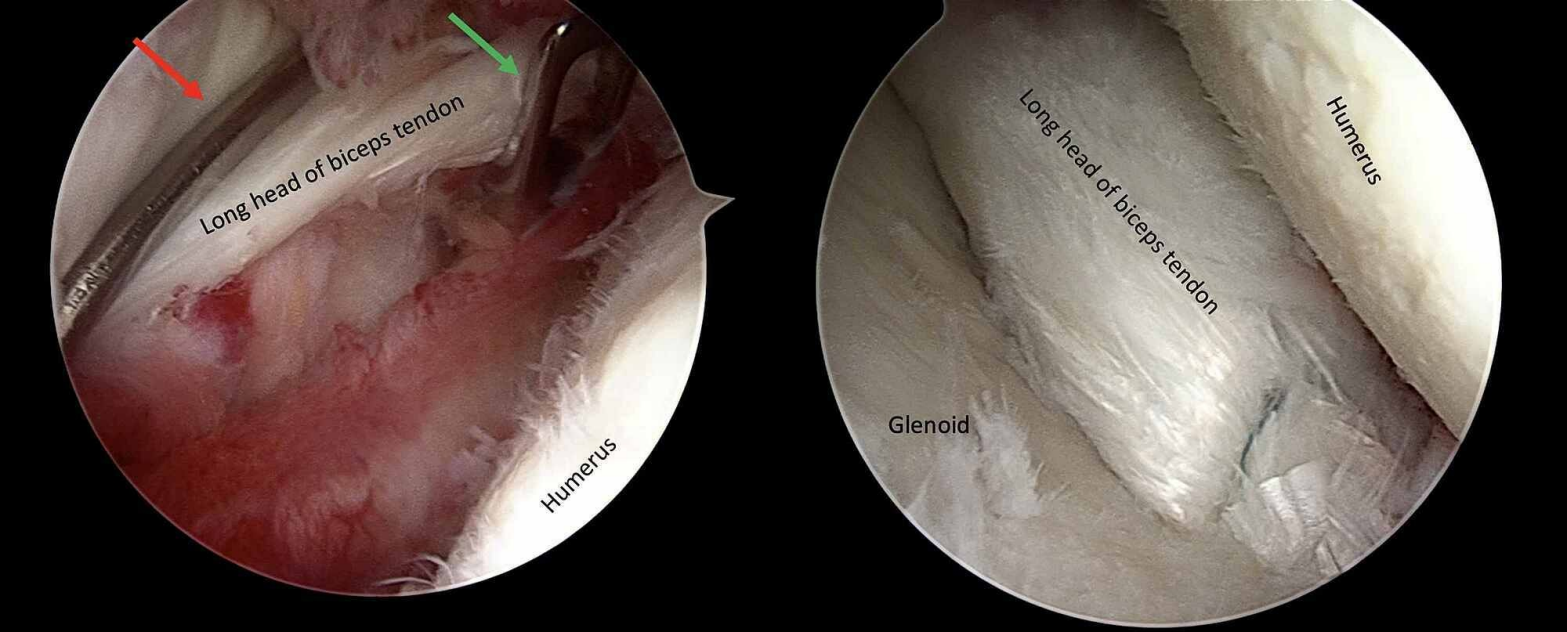
Intraoperative view of case 1 (a) Pressure onto LHBT with a probe (red arrow) and cut with a punch (green arrow) and (b) an intraarticular view of labral reconstruction. LHBT: long head of the biceps tendon.

The patient underwent shoulder arthroscopy. During surgery, it was decided that the anterior labrum was defective (Franceschi type III) and could not be repaired (Figure 3a). Neither Latarjet procedure was planned, as there was no GBL, nor reverse shoulder arthroplasty was planned, as the patient was relatively young. It was decided to use the LHBT in the reconstruction of the labrum (Figure 4b). The distal part of the LHBT was set free and biceps tenotomy was performed due to the patient's age.

**Figure 5:**
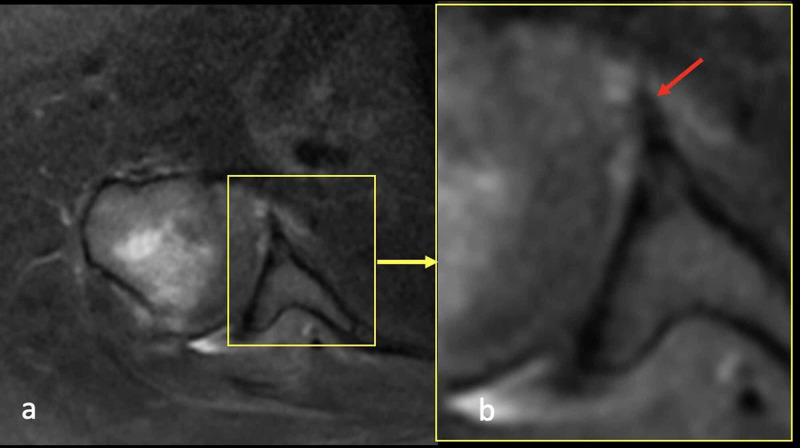
MRI images of case 1 (a) An axial image showing totally fixed LHBT to the labrum; (b) a magnification view of MRI showing the LHBT in the glenoid (red arrow). LHBT: long head of the biceps tendon.

**Figure 6:**
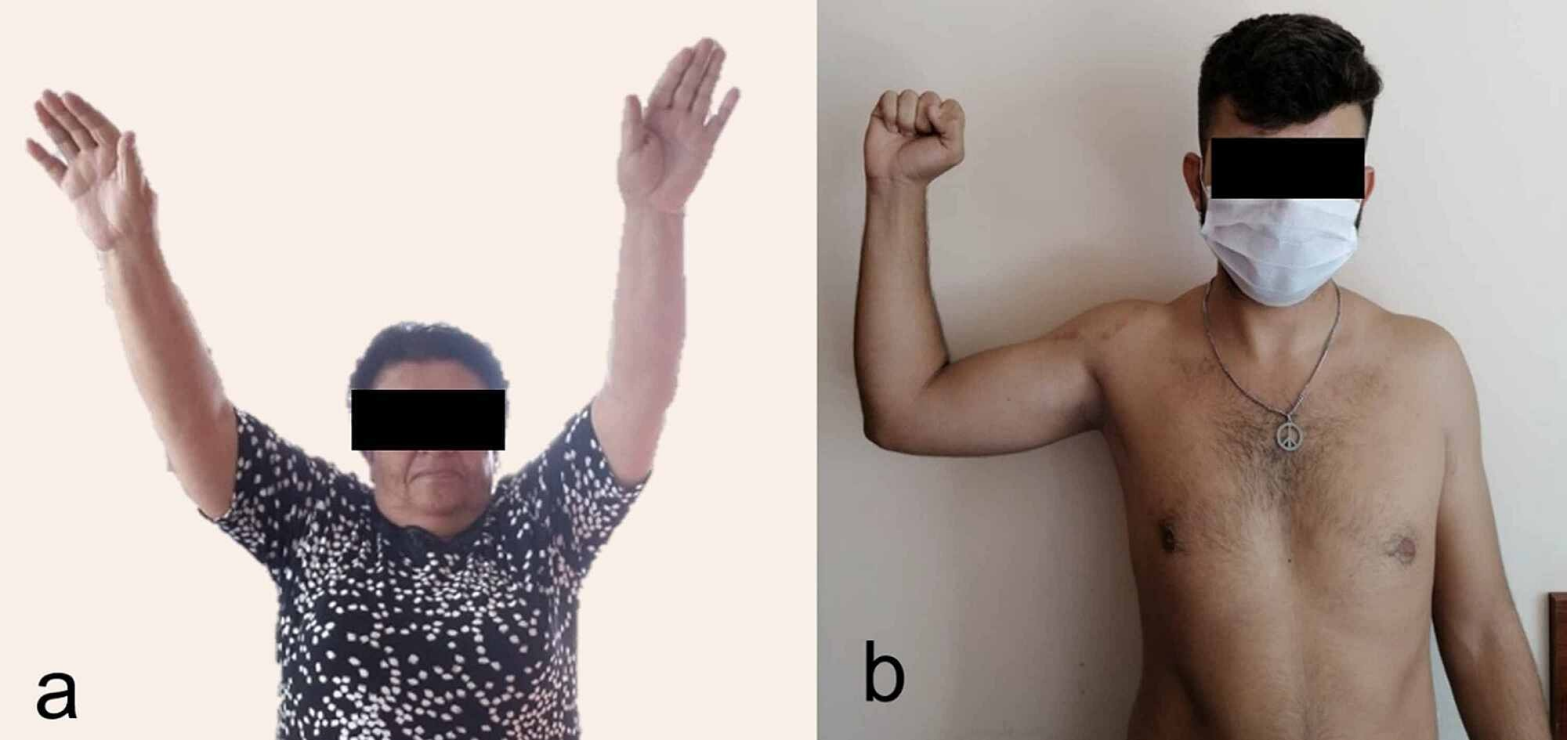
Postoperative clinical images of cases (a) Case 1 and (b) case 2.

The patient was instructed to wear a shoulder sling for three weeks. After three weeks, passive shoulder movements and pendular movements were initiated. Active shoulder movements were allowed at five weeks. Active and passive elbow movements were allowed immediately after surgery. At six months of follow-up, MRI showed the LHBT was fixed to the labrum completely (Figure 5). The Constant Shoulder Score (CSS) was 92 (Figure 6a).

Case 2

A 17-year-old male was admitted to our clinic with a history of more than 10 shoulder dislocations during the last two years. The patient described utilizing a self-reduction technique to relocate the articulation. He had no prior shoulder injuries before the time of trauma. The apprehension test and fear test were positive. The MRI images revealed a Hill-Sachs and labral tear. No GBL was detected on either CT or MRI. After the diagnosis of Bankart lesion, the arthroscopic labral repair was planned. Written informed consent was obtained from the patient.

**Figure 7:**
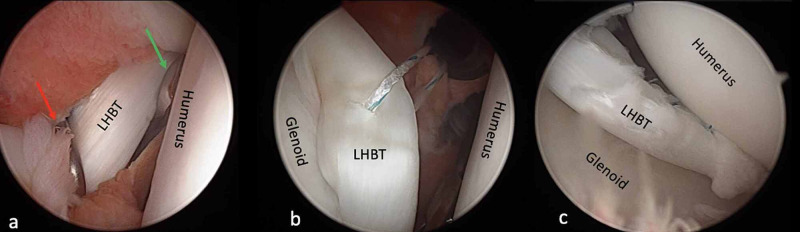
Intraoperative views of case 2 (a) Pressure onto LHBT with a probe (red arrow) and cut with a punch (green arrow); (b) the anchor was placed at the level of 3 o’clock, and labral reconstruction was applied; (c) postoperative view of reconstruction. LHBT: long head of the biceps tendon.

The patient underwent routine shoulder arthroscopy. During surgery, it was decided that the anterior labrum was defective (Franceschi type III) and could not be repaired (Figure 3b). Therefore, the Duru technique was done (Figure 7). Following surgery, active elbow exercises were started after three weeks due to biceps tenodesis. The patient was instructed to wear a shoulder sling for three weeks. After three weeks, passive shoulder movements and pendular movements were initiated. Active shoulder movements were allowed at five weeks.

**Figure 8:**
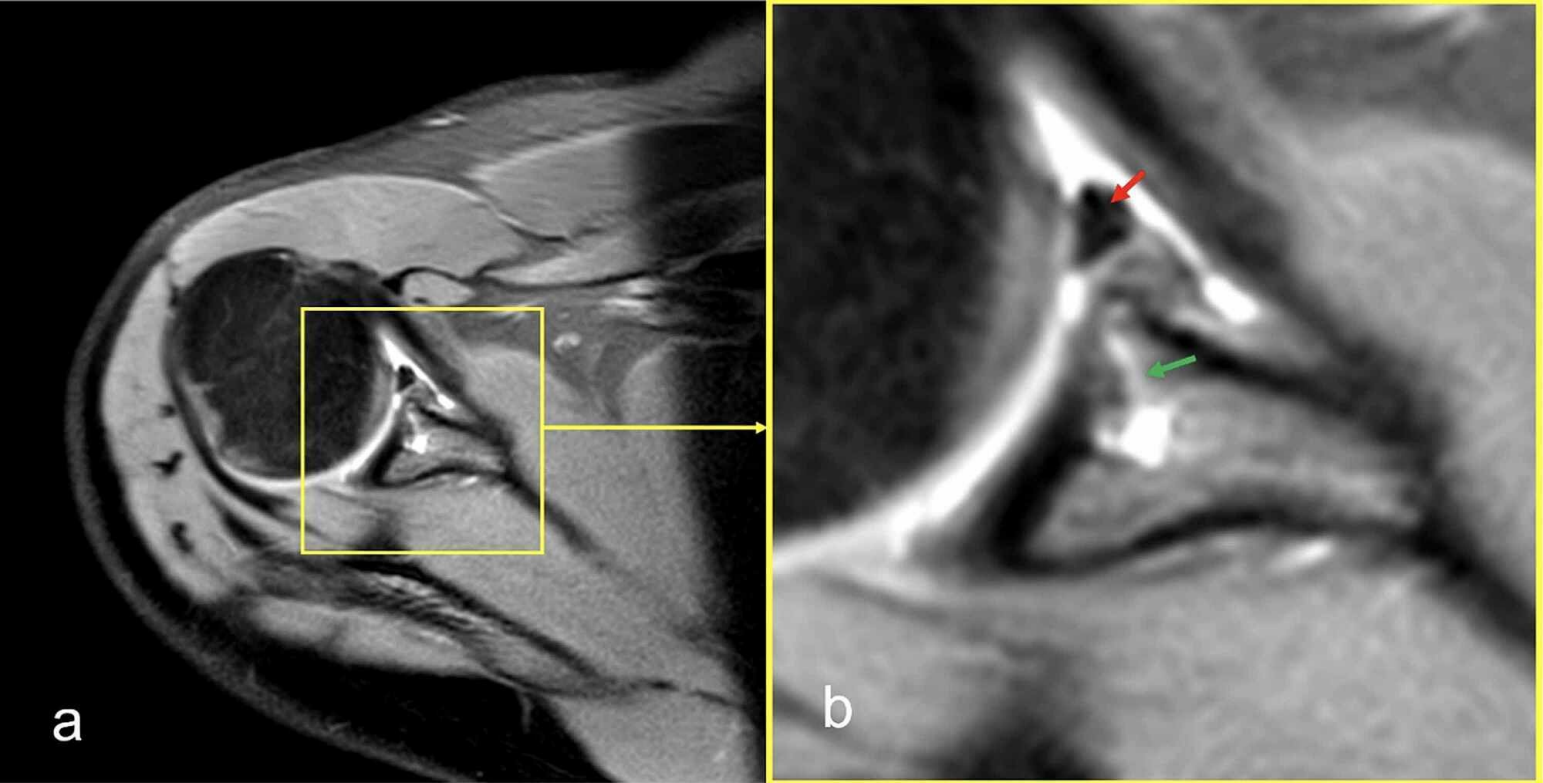
Postoperative MRI images of case 2 (a) An axial image showing totally fixed LHBT to glenoid; (b) a magnification view of MRI showing the LHBT in the glenoid (red arrow) and anchor (green arrow). LHBT: long head of the biceps tendon.

At six months of follow-up, MRI showed the LHBT was tightly fixed to the glenoid (Figure 8). The CSS was 100 (Figure 6b).

## Discussion

After an anterior shoulder dislocation, anatomic structures that stabilize the joint are damaged and weakened including ligaments, capsule, labrum, and glenoid bone. However, the degree of damage in these tissues varies depending on the clinical characteristics of each patient. A limited number of cases presents with normal glenoid without repairable labral tissue. This is also the case for revision cases. In this group of patients, the Duru technique may be a useful method.

The poor capsulolabral quality is one of the risk factors for failure after primary or revision arthroscopic Bankart repair. Several authors have recommended different techniques for the management of such cases. Causes of failure after primary and revision instability operations include incorrect instability diagnosis, technical operation errors, young-aged patients, contact sports, traumatic events, bone loss at the glenoid or humeral side, or poor capsulolabral quality [7-9].

If the labral tissue is severely damaged or no labral tissue exists, particularly in revision Bankart cases with a bone defect <25% or those with defective labrum and no bone defect, soft tissue techniques are applied, such as rotator interval narrowing, subscapular augmentation, remplissage or capsular plication, or bone surgery techniques such as Latarjet, in addition to labral repair to provide a stable glenohumeral joint [7-9]. However, for those with revision anterior instability and no GBL, or primary patients in whom there is insufficient labral tissue, no gold-standard soft tissue operation has been established, yet.

In a study, Randelli et al. [3] showed that the lack of external rotation developed in patients after rotator interval closure (RIC). In a systematic review by Bozzo et al. [4], it was concluded that the indications for RIC were poorly reported, and the surgical techniques were inconsistent. Although most studies have reported positive clinical results, the heterogeneity of outcome measures limits the inference of definitive statements about which types of RIC are warranted for selected subgroups undergoing arthroscopic shoulder stabilization [4].

In a series of 110 patients, Maiotti et al. [5] applied arthroscopic subscapularis augmentation. This procedure was shown to be effective in restoring joint stability in patients engaged in sports, those affected by chronic anterior shoulder instability associated with anterior GBL (<25%), capsular deficiency, and Hill-Sachs lesions, with mild restriction of external rotation. In addition, remplissage and capsular plication could lead to external rotation limitation [6].

The success of the revision repair depends on sufficient re-tensioning of the capsulolabral complex and adequate incorporation of the IGHL, which is the main restraint against anterior translation in the abduction and external rotation, and most recurrent anterior instabilities occur in this joint position [7-9]. Although labral reconstruction with tendon transfer by benefitting from the sling effect of the LHBT has been reported in the literature, there may be a risk of axillary nerve damage and pain associated with biceps tendon transfer [10,11].

The Duru technique can be considered for use in patients with a poor capsulolabral quality. In this technique, we attempted to obtain an anatomic or close to an anatomic structure by recreating the labral tissue with LHBT. Moreover, by suturing the IGHL to the LHBT at the 5 o’clock level, capsule tension can be adjusted, as desired. With the sacrifice of the biceps tendon, the loss of functions of this tendon can be overcome with biceps tenodesis. By cutting the LHBT at the desired length, sufficient tissue can be obtained for labral reconstruction. Of note, to apply the Duru technique, there must be an absolute evaluation of whether there is GBL. Other landmarks guiding the treatment are the evaluation of the size and location of the Hill-Sach lesion, the presence of a humeral avulsion of the glenohumeral ligament lesion, ligamentous laxity, and tissue quality of the labrum and capsule. This technique can be a useful option, particularly in cases where the labral tissue has been lost or the labrum has thinned in primary or revision surgery. However, it should be kept in mind that each individual case is different, and therefore, patient-specific treatment protocols should be planned. In patients with a GBL of <25% and Franceschi types II and III labral defects with primary or revision anterior glenohumeral instability, labral reconstruction with the Duru technique may be applied.

Although the evidence is only based on two cases, in the light of the experience gained from this case, without making explicit statements, the Duru technique can be considered to have some advantages and disadvantages.

The major advantages are: (i) it can be used as the primary procedure in patients with a labral defect or in revision surgery in patients with a GBL of <25%; (ii) it can be used as the first option in patients with shoulder instability with anatomic variations such as the Buford complex; (iii) it is easier, safer, and less invasive than arthroscopic Latarjet; (iv) it does not cause external rotation limitation; (v) with a cut from the biceps muscle bicipital groove, a length can be obtained from the LHBT that will wrap 360° around the labrum. This technique can be used in selected patients with anterior and posterior labral defects.

Disadvantages are: (i) no long-term follow-up results are available; (ii) it has not been extensively studied, yet; (iii) the results of this technique are based on short-term follow-up results of two cases.

## Conclusions

In conclusion, this technique can be a useful option, particularly in cases where the labral tissue has been lost or thin in primary or revision surgery. Additionally, length can be obtained from the LHBT that will wrap 360° around the labrum thus posterior labrum can be reconstructed if needed.

Reconstruction with the LHBT is a feasible option in the management of patients with recurrent instability and subcritical GBL and may be considered as an alternative to the Latarjet procedure in this patient population. Nonetheless, further clinical and biomechanical studies are needed to compare this procedure to more commonly utilized techniques.
